# Tertiary lymphoid structures accompanied by fibrillary matrix morphology impact anti-tumor immunity in basal cell carcinomas

**DOI:** 10.3389/fmed.2022.981074

**Published:** 2022-10-27

**Authors:** Candice Byers, Melissa Gill, Nicholas R. Kurtansky, Christi Alessi-Fox, Maggie Harman, Miguel Cordova, Salvador Gonzalez, Pascale Guitera, Veronica Rotemberg, Ashfaq Marghoob, Chih-Shan Jason Chen, Jennifer Dy, Kivanc Kose, Milind Rajadhyaksha, Aditi Sahu

**Affiliations:** ^1^The Institute for Experiential AI, Roux Institute, Northeastern University, Portland, ME, United States; ^2^Department of Clinical Pathology and Cancer Diagnostics, Karolinska University Hospital, Stockholm, Sweden; ^3^Department of Pathology, SUNY Downstate Health Sciences University, Brooklyn, NY, United States; ^4^Faculty of Medicine and Health Sciences, University of Alcala de Henares, Madrid, Spain; ^5^Memorial Sloan Kettering Cancer Center, New York, NY, United States; ^6^Caliber Imaging and Diagnostics, Rochester, NY, United States; ^7^Sydney Melanoma Diagnostic Center, Royal Alfred Prince Hospital, Camperdown, NSW, Australia; ^8^Melanoma Institute Australia, Sydney, NSW, Australia; ^9^Department of Electrical and Computer Engineering, Northeastern University, Boston, MA, United States; ^10^The Institute for Experiential AI, Northeastern University, Boston, MA, United States

**Keywords:** basal cell carcinoma (BCC), tertiary lymphoid structures (TLS), extracellular matrix (ECM), tumor infiltrating lymphocyte (TIL), fibrillary morphology, collagen, mucin, tumor microenvironment

## Abstract

Tertiary lymphoid structures (TLS) are specialized lymphoid formations that serve as local repertoire of T- and B-cells at sites of chronic inflammation, autoimmunity, and cancer. While presence of TLS has been associated with improved response to immune checkpoint blockade therapies and overall outcomes in several cancers, its prognostic value in basal cell carcinoma (BCC) has not been investigated. Herein, we determined the prognostic impact of TLS by relating its prevalence and maturation with outcome measures of anti-tumor immunity, namely tumor infiltrating lymphocytes (TILs) and tumor killing. In 30 distinct BCCs, we show the presence of TLS was significantly enriched in tumors harboring a nodular component and more mature primary TLS was associated with TIL counts. Moreover, assessment of the fibrillary matrix surrounding tumors showed discrete morphologies significantly associated with higher TIL counts, critically accounting for heterogeneity in TIL count distribution within TLS maturation stages. Specifically, increased length of fibers and lacunarity of the matrix with concomitant reduction in density and alignment of fibers were present surrounding tumors displaying high TIL counts. Given the interest in inducing TLS formation as a therapeutic intervention as well as its documented prognostic value, elucidating potential impediments to the ability of TLS in driving anti-tumor immunity within the tumor microenvironment warrants further investigation. These results begin to address and highlight the need to integrate stromal features which may present a hindrance to TLS formation and/or effective function as a mediator of immunotherapy response.

## Introduction

Tertiary lymphoid structures (TLS) are ectopic, vascularized lymphoid formations that arise in non-lymphoid tissues during chronic inflammation, such as in autoimmune disease, chronic infection, and cancer (1). Although TLS are not found under physiological conditions in nonlymphoid tissues, they structurally resemble secondary lymphoid organs such as lymph nodes (2). Depending on their maturation stage, TLS are frequently characterized by the presence of B-cell aggregates, germinal B-cell centers, dendritic cells and T-cells in association with high endothelial venules (HEVs). In these non-lymphoid tissues, TLS serve as specialized sites for initiation and maintenance of local and systemic B- and T-cell responses. In fact, tumor-specific T- and B-cell immunity serve as potentiating stimuli for TLS formation within the tumor microenvironment (TME), and heterogeneity in driving mechanisms result in variable TLS maturation stages (e.g., aggregate, primary, secondary) (1).

Overall, TLS presence is associated with a reduced risk of recurrence, prolonged and disease-free survival, and improved response to immune checkpoint blockade (ICB) in several cancers (3). TLS are also frequently correlated with density of tumor-infiltrating lymphocytes (TILs) in solid tumors (4). Tumors with mature TLS, a high density of B cells and plasma cells, as well as antibodies to tumor-associated antigens are typically associated with favorable clinical outcomes and immunotherapy response (5, 6). Based on prognostic effects of TLS in cancers, they are heralded as potential mediators of anti-tumor immunity. In oral squamous cell carcinoma (OSCC), TLS maturation correlated with increased intratumor cytotoxic T and NK cell densities (7). Several studies have investigated the importance of TLS in melanoma (8–11) and also cutaneous squamous cell carcinoma (SCC) (12), however, the contributions of TLS to intratumoral immune response and the interactions between the TLS and the extracellular matrix (ECM) remain to be understood.

Drastic remodeling of the extracellular matrix (ECM) occurs during the progression of tumor growth thereby replacing normal ECM with tumor-associated ECM (13–16). Studies have associated remodeling of the ECM with survival outcomes in various cancers, however mechanisms underlying how these changes directly affect survival or response to therapy remain elusive with some studies reporting conflicting outcomes (17–20). Regardless these studies are suggestive of an immunomodulatory role for specific ECM components, in addition to a physical barrier to immune cells (21).

Most studies are focused on attributing alterations in ECM with modulating cancer cell behavior whereas less emphasis has been given to immunomodulatory roles. The capability for components of the ECM to affect immune cell activity has recently been documented, although largely for more abundant fibrillary matrix components, such as collagen type I (22–24). Other sources of reconfiguring tumor-associated ECM to confer tumor evasion of immune infiltration has been attributed to both tumor cells and recently cancer-associated fibroblasts (25–27). Less abundant components of the ECM (such as reticulin and elastin) that comprise the fibrillary matrix along with alterations associated with disease (such as deposition of amyloid and mucin) currently have less defined roles in directly or indirectly modulating immune cell behavior (28). Some studies have shown cancer-associated secreted mucins play an important role in inducing overall immunosuppression through several mechanisms such as creation of a physical barrier, prevention of cancer cell recognition and inhibition of cytolytic activity (29).

In skin cancer, a mechanistic role for collagen has been described toward facilitating tumor cell invasiveness (30–32). In *in vitro* studies, an immunosuppressive role has been attributed to increased collagen density that leads to downregulating effector T cell activity and upregulating T-regulatory cell markers. Further, impaired cytotoxic activity of these T cells was confirmed when co-cultured with melanoma cells (33). Studies directed at ECM degradation have shown to improve immunotherapy response in preclinical models for melanoma (34, 35). While targeting the ECM may prove to be a promising approach to improve response to therapy, the mutual interdependence of ECM on various processes in the TME complicates interpretation. Therefore, discrete immune modulatory functions of specific components of the ECM and their influence on anti-tumor immunity (i.e., TILs) should be mechanistically resolved to tailor ECM-targeting and better predict outcomes of cancer therapies (13).

In this study, we investigated the presence, cellular composition, and maturation stage of TLS within peritumoral regions of BCC tumors and how they relate to anti-tumor immunity. Tumor-infiltrating lymphocytes (TILs) and tumor killing were used as outcome measures to assess functional effect of TLS in anti-tumor immunity. The relationship between TLS and TILs was further investigated by quantifying stromal fibrillary morphology within the ECM along with presence of peritumoral mucin and/or amyloid.

## Materials and methods

### Patient and tissue details

Patients with primary diagnosis of BCC (no prior biopsy or treatment) at Dermatology Service at Memorial Sloan Kettering Cancer Center (MSKCC), New York were identified and consecutively recruited. Specific anatomic locations were then assigned to one of the following general body sites: head and neck (sun-exposed), trunk, extremity (patient characteristics and lesion details are provided in Table 1).

**Table 1:** Patient and lesion characteristics.

**Patient _ID**	**Age**	**Gender**	**Therapy**	**Lesion_ID**	**Body site (location) ^∧^= sun-exposed**	**Diagnosis**	**Superficial(s)**	**Nodular (n)**	**Infiltrative(i)**	**Ulceration**
Patient 1	43	F	no	Patient 1_ lesion 1	Trunk (upper back)	BCCs	Yes	No	No	Yes
Patient 2	73	F	no	Patient 2_ lesion 2	Trunk (abdomen)	BCCn	No	Yes	No	Yes
			no	Patient 2_ lesion 3	Trunk (chest)	BCCn	No	Yes	No	Yes
Patient 3	88	F	no	Patient 3_ Lesion 4	Head and neck^∧^(forehead)	BCCn	No	Yes	No	No
Patient 4	64	F	no	Patient 4_ lesion 5	Head and neck^∧^(lip)	BCCs	Yes	No	No	Yes
Patient 5	71	M	no	Patient 5_ lesion 6	Head and neck^∧^(cheek)	BCCn	No	Yes	No	No
Patient 6	66	M	no	Patient 6_ lesion 7	Head and neck^∧^(posterior neck)	BCCsn	Yes	Yes	No	Yes
Patient 7	75	M	no	Patient 7_ lesion 8	Extremity (shin)	BCCn	No	Yes	No	Yes
Patient 8	66	M	no	Patient 8_ lesion 9	Extremity (proximal arm)	BCCs	Yes	No	No	Yes
			no	Patient 8_ lesion 10	Trunk (back)	BCCsn	Yes	Yes	No	Yes
			no	Patient 8_ lesion 11	Head and neck^∧^(forehead)	BCCn	No	Yes	No	Yes
			no	Patient 8_ lesion 12	Head and neck^∧^(lip)	BCCn	No	Yes	No	Yes
			no	Patient 8_ lesion 13	Head and neck^∧^(nasal bridge)	BCCs	Yes	No	No	Yes
Patient 9	83	F	no	Patient 9_ lesion 14	Head and neck^∧^(cheek)	BCCni	No	Yes	Yes	No
Patient 10	59	M	no	Patient 10_ lesion 15	Trunk (abdomen)	BCCn	No	Yes	No	No
			no	Patient 10_ lesion 16	Trunk (chest)	BCCn	No	Yes	No	No
Patient 11	81	M	no	Patient 11_ lesion 17	Head and neck^∧^(lip)	BCCn	No	Yes	No	Yes
Patient 12	74	F	no	Patient 12_ lesion 18	Trunk (lower back)	BCCn	No	Yes	No	No
			no	Patient 12_ lesion 19	Trunk (clavicle)	BCCn	No	Yes	No	Yes
Patient 13	N/A	N/A	no	Patient 13_ lesion 20	Head and neck^∧^(nose)	BCCsni	Yes	Yes	Yes	Yes
Patient 14	72	M	no	Patient 14_ lesion 21	Head and neck^∧^(cheek)	BCCni	No	Yes	Yes	Yes
Patient 15	90	M	no	Patient 15_ lesion 22	Head and neck^∧^(nose)	BCCn	No	Yes	No	Yes
Patient 16	51	M	no	Patient 16_ lesion 23	Trunk (upper back)	BCCsn	Yes	Yes	No	Yes
Patient 17	64	M	no	Patient 17_ lesion 24	Extremity (shoulder)	BCCsn	Yes	Yes	No	Yes
Patient 18	62	M	no	Patient 18_ lesion 25	Head and neck^∧^(chin)	BCCn	No	Yes	No	Yes
			no	Patient 18_ lesion 26	Head and neck^∧^(neck)	BCCni	No	Yes	Yes	Yes
Patient 19	53	F	no	Patient 19_ lesion 27	Head and neck^∧^(forehead)	BCCn	No	Yes	No	No
Patient 20	80	F	no	Patient 20_ lesion 28	Head and neck^∧^(nose)	BCCi	No	No	Yes	Yes
Patient 21	68	M	no	Patient 21_ lesion 29	Trunk (chest)	BCCn	No	Yes	No	No
Patient 22	54	M	no	Patient 22_ lesion 30	Trunk (upper back)	BCCsn	Yes	Yes	No	No

Serial sections were acquired for a different set of experiments from FFPE blocks of primary biopsies (not excisions). Only the first section was submitted for H&E while other sections were used for immunohistochemistry (IHC). TLS and TILs were analyzed on dual IHC stained CD3/CD20 sections while ECM features were analyzed on corresponding H&E sections (not serial sections).

### Dual CD3/CD20 IHC

Multiplex IHC for detection of both CD3 and CD20 was performed on an automated staining platform, BOND RX Autostainer (Leica Biosystems, Germany). IHC procedure using BOND RX included heat retrieval, incubation with primary antibodies (primary anti-CD3, Santa Cruz, 1:200 dilution, catalog#: NCL-L-CD3-565 and primary anti-CD20, Dako Agilent, 1:3,000 dilution, catalog#: M0755) followed by polymer detection kits (Leica Biosystems, catalog# DS9390, DS9800). The IHC slides were digitized on slidescanner (Aperio Imagescope, Leica Biosystems, US) for quantitative analysis.

### TLS annotation and evaluation of maturation stage

TLS presence was defined as T- and B-cell clusters surrounding high endothelial venules on dual CD3/CD20 IHC stained sections (3). TLS area was annotated by a reader followed by QC by a board-certified dermatopathologist (MG). TLS were sequentially numbered and the annotations were used to compute size, total number of cells, and B cell fraction. Tumors were also annotated on IHC sections to measure tumor area and infiltrating T and B cells, henceforth referred to as TILs. Tumor within 500 μm from the edge of TLS was inferred as “proximal tumor” while tumor farthest from TLS on the same section, or on a serial section was inferred as “distal tumor”. These annotated tumors were tagged with TLS number so their numbering reflected their respective TLS (e.g., TLS1_tumor-proximal1, TLS2_tumor-proximal2). For lesions lacking TLS, a randomly selected field-of-view was used to obtain representative tumor within a 500 x 500 μm^2^ area (referred to as “control” tumor). Total tissue area was also measured on the IHC sections to estimate relative TLS size.

For the fibrillary matrix, stromal region surrounding tumor was annotated on the corresponding H&E section. A 30 μm area perimeter from the tumor edge was used to capture fibrillary morphology features. QC on annotated tumor and stromal areas was done by the dermatopathologist.

Additionally, the annotated TLS and tumor area on the IHC sections was reviewed for TLS maturation staging (aggregate, primary or secondary) and tumor killing, respectively, by the dermatopathologist. Previously published criteria were used to define TLS maturation stages as follows:

Aggregate TLS-dense lymphocytic aggregates composed primarily of T-cells with randomly distributed B-cells; primary TLS: B-cell clusters with T-cells and follicular dendritic cells but lacking germinal center; secondary TLS: B-cell clusters with germinal center-like maturation surrounded by T-cells, and evenly dispersed follicular dendritic cells (11, 36–38). Tumor killing (used analogous to active regression) was used as another outcome measure since it is indicative of an active anti-tumor immune response. We defined tumor killing or active regression as a combination of immune infiltration and disruption of the palisaded architecture of the tumor cells at the periphery, with or without occurrence of apoptotic cells, and dermal deposition of collagen (39). Binary assessment for tumor killing presence/absence was evaluated by the dermatopathologist, along with presence/absence of mucin and amyloid on the corresponding H&E section.

### Lymphocyte quantification

Each immune marker was quantified using Positive Pixel counting algorithm (Aperio, Leica Biosystems, US). Thresholding was performed on brown, pink, and total brown and pink areas. The total area was determined by hematoxylin-stained area. Integrated positive pixel area was used to compute the relative proportion of cells. Parameters for threshold, hue, and saturation were kept constant across all patient specimens.

### Isolation of tumor-associated extracellular matrix

Annotated H&E images were used to generate tumor and stroma masks using Seg3D2 (https://www.sci.utah.edu/cibc-software/seg3d.html). Tumor region was subtracted from stroma to extract stroma margins (using 30 μm area perimeter described above). H&E images of isolated stroma from individual tumors were then deconvoluted in Fiji to separate eosin and hematoxylin channels (40). Resulting eosin images were further labeled for specific components of the ECM (amyloid, mucin, fibrillary matrix) as well as nuclei to train a segmentation model using the Weka segmentation plugin in Fiji (41). The segmentation model resulting from training classifiers for discrete ECM components and nuclei were then applied to all eosin images (*n* = 68) (Supplemental Figure S2A). The labels for fibrillary matrix include features representing collagen, elastin, and reticulin, which were unable to be distinctly segmented using H&E and are therefore combined as a single classifier referred to as “fibrillary matrix”. Based on the classifiers trained in the Weka segmentation model, we generated probability maps for each class and used the fibrillary matrix segmentation as input in Fiji plugin Twombli to quantify matrix patterns (42). Images of fibrillary matrix were deemed ineligible for analysis with Twombli when not meeting image quality criteria such as blurriness or reduced resolution (referring to default parameters in Twombli documentation). Different models for Weka segmentation were trained and tested under the guidance of board-certified dermatopathologist (MG) to develop accurate segmentation of fibrillary matrix from H&E images (Supplemental Figure S2D).

### Congruency between tumor boundaries in IHC and H&E to associate fibrillary matrix morphologies with TIL counts

The CD3/CD20 IHC and corresponding H&E sections were not serial sections, thus few lesions where no tumor correlate was identified in IHC were excluded from analysis. Further, it also led to discrepancies when the fibrillary matrix mask generated on H&E was overlaid on the IHC section. In some cases, tumor boundary interrupts stromal masks, generating more than one stromal region per tumor. These multiple stromal regions were combined to encompass a single stromal measurement per tumor. Each measurement from Twombli analysis either is an additive measurement of individual fibers, such as number of endpoints or branchpoint, or a quantification of patterns, such as curvature or alignment. For instances where individual fibers are added up for a measurement, these measurements were added to combine stromal regions whereas global patterns were combined using a weighted average based on area of fibrillary mask.

### Statistical analysis

#### Prevalence of TLS in BCC lesions

The outcome of interest for the lesion-level analysis (Table 2) is the binary presence of TLS. A secondary outcome was the binary presence of *primary* TLS in the excised specimen (secondary TLS were not found in this dataset, only aggregate and primary were encountered). The extent of association between the outcome and various categorical factors (namely body site, gender, and histological subtype and maturation stage of TLS) were quantified using odds ratios with 95% confidence intervals computed using the Wald method and statistical significance of each association was assessed *via* Fisher's exact test. The association between the outcome and patient age was assessed using the Student's T-test after first confirming the age distributions were approximately Gaussian.

**Table 2:** Prevalence of TLS in BCC lesions.

	**Overall Lesions**	**Any TLS identified in primary biopsy**				**Primary TLS identified in primary biopsy**			
		**TRUE**	**FALSE**			**TRUE**	**FALSE**		
**Subsample**	*N (col %)*	*N* *(row prop)*	*N (row prop)*	OR (95%)**	p-value*	*N* *(row prop)*	*N (row prop)*	OR (95% CI)**	p-value*
*Total*	30 (1)	19 (0.63)	11 (0.37)	–	–	11 (0.37)	19 (0.63)	–	–
**Body Site**
Trunk	11 (0.37)	7 (0.64)	4 (0.36)	1.05 (0.21–5.16)	1.000	3 (0.27)	8 (0.73)	0.48 (0.09–2.52)	0.448
Extremity	3 (0.1)	2 (0.67)	1 (0.33)	1.20 (0.09–29.14)	1.000	1 (0.33)	2 (0.67)	0.64 (0.05–8.62)	1.000
Head and neck	16 (0.53)	10 (0.63)	6 (0.38)	1.00 (referent)	–	7 (0.44)	9 (0.56)	1.00 (referent)	–
**Gender**
Female	10 (0.33)	4 (0.4)	6 (0.6)	0.24 (0.05–1.21)	0.114	2 (0.2)	8 (0.8)	0.34 (0.06–2.07)	0.414
Male	19 (0.63)	14 (0.74)	5 (0.26)	1.00 (referent)	–	8 (0.42)	11 (0.58)	1.00 (referent)	–
Unknown	1 (0.03)	1 (1)	0 (0)	–	–	1 (1)	0 (0)	–	–
**Infiltrative**
Present	5 (0.17)	3 (0.6)	2 (0.4)	0.84 (0.12–6.03)	1.000	3 (0.6)	2 (0.4)	3.19 (0.44–23.01)	0.327
Absent	25 (0.83)	16 (0.64)	9 (0.36)	1.00 (referent)	–	8 (0.32)	17 (0.68)	1.00 (referent)	–
*Nodular*									
Present	25 (0.83)	18 (0.72)	7 (0.28)	10.29 (0.97–108.81)	0.047	10 (0.4)	15 (0.6)	2.67 (0.26–27.49)	0.626
Absent	5 (0.17)	1 (0.2)	4 (0.8)	1.00 (referent)	–	1 (0.2)	4 (0.8)	1.00 (referent)	–
**Superficial**
Present	10 (0.33)	6 (0.6)	4 (0.4)	0.81 (0.17–3.86)	1.000	4 (0.4)	6 (0.6)	1.24 (0.26–5.91)	1.000
Absent	20 (0.67)	13 (0.65)	7 (0.35)	1.00 (referent)	–	7 (0.35)	13 (0.65)	1.00 (referent)	–
*Ulceration*									
TRUE	21 (0.7)	11 (0.52)	10 (0.48)	0.14 (0.01–1.30)	0.100	6 (0.29)	15 (0.71)	0.32 (0.06–1.62)	0.225
FALSE	9 (0.3)	8 (0.89)	1 (0.11)	1.00 (referent)	–	5 (0.56)	4 (0.44)	1.00 (referent)	–
*Mean Age (IQR)*	67.9 (62,74)	67.3 (62,72)	68.9 (61,77)	*N/A*	0.713 ***	67.5 (60,71)	68.11 (63,74)	*N/A*	0.893 ***
		*1 unknown age*				*1 unknown age*			

* Fisher's exact test.

** Wald method.

*** t-test.

#### Spatial effect of TLS on TILs for individual tumors

The unit of analysis are individual tumors relative to the nearest TLS and its maturation stage (tumor-level analysis). The outcome of interest are TIL counts (Table 3). The primary independent variable is the tumor proximity to TLS and its maturation stage. We present mean TIL counts across the primary independent variable as well as covariates, namely body site of lesion, gender, age, BCC subtype and TLS maturation stage. Significance in the differences of Box-Cox transformed TIL counts are modeled using mixed-effects models with a random intercept defined for each BCC containing TLS (*n* = 19). *P-*values are derived from Wald tests of the fixed effect in the models.

**Table 3:** Effect of TLS maturation stage and proximity to BCC on TIL count (*n* represents individual tumors).

		**Tumors proximal to primary TLS (A)**		**Tumors proximal to aggregate TLS (B)**		**Tumors with no evidence of TLS (C)**		***p-*** **value**		
		***n***	**Mean TIL count (IQR)**	***n***	***Mean TIL count (IQR)***	***n***	***Mean TIL count (IQR)***	***A to B***	***A to C***	***B to C***
**Total Tumors**		48	38.1 (4.78,44.1)	162	17.6 (1.68,20.2)	49	7.98 (0.85,6.71)	<0.001	0.118	0.83
**Location**
	Head and neck	37	35.6 (4.87,40.1)	85	15.5 (1.43,17)	41	4.13 (0.68,4.11)	<0.001	0.06	0.506
	Trunk	7	65 (20.7,101)	46	25.1 (1.86,22.6)	7	27.8 (7.73,31.9)	0.132	0.189	0.536
	Extremity	4	14 (1.79,14.8)	31	12.2 (1.59,13.3)	1	26.8 (26.8,26.8)	0.772	-	-
	*p-*value (h&n vs. trunk)		0.190		0.454		0.094			
	*p-*value (h&n vs. extremity)		0.265		0.967		0.274			
**Gender**
	Male	43	36.1 (5.02,45.4)	132	19.4 (1.74,22.2)	11	16.1 (5.77,24.7)	<0.001	0.719	0.497
	Female	4	64.7 (4.19,66.2)	19	6.66 (0.86,5.14)	38	5.63 (0.58,3.89)	0.545	0.349	0.995
	Unknown	1	–	11	–	0	–	–	–	–
	*p-*value		0.924		0.576		0.248			
**Age**
	43–65	11	55.5 (6.28,53.4)	46	21.4 (1.86,21.5)	8	21.5 (5.74,20.4)	0.186	0.584	0.689
	66–90	36	33.4 (4.78,40.8)	105	16.2 (1.36,15.8)	41	5.34 (0.68,4.11)	<0.001	0.18	0.675
	Unknown	1	–	11	–	0	–	–	–	–
	*p-*value		0.579		0.725		0.333			
**Infiltrative component**
	Absent	29	37.1 (4.15,40.1)	135	19.3 (1.86,21.4)	30	10.8 (0.91,11)	0.239	0.197	0.842
	Present	19	39.6 (6.01,50.7)	27	9.33 (0.89,9.25)	19	3.56 (0.88,2.62)	<0.001	0.808	0.826
	*p-*value		0.548		0.213		0.799			
**Nodular component**
	Absent	4	16.2 (7.64,17.7)	1	96.8 (96.8,96.8)	20	7.96 (0.92,3.22)	–	0.966	–
	Present	44	40.1 (4.42,48.5)	161	17.1 (1.66,18.9)	29	7.99 (0.85,11.4)	<0.001	0.129	0.871
	*p-*value		0.749		0.211		0.799			
**Superficial component**
	Absent	37	43.5 (4.87,48.1)	102	20.8 (1.33,21.8)	45	5.03 (0.8,4.56)	<0.001	0.041	0.444
	Present	11	20 (5,32.9)	60	12.2 (2.02,16)	4	41,1 (21.9,50.1)	0.372	0.293	0.105
	*p-*value		0.483		0.987		0.079			
**Ulceration**
	FALSE	11	64.9 (8.05,101)	40	25.3 (1.22,19.8)	14	1.23 (0.26,1.78)	0.109	0.032	0.197
	TRUE	37	30.1 (4.15,40.1)	122	15.1 (1.86,19.8)	35	10.7 (1.31,13.3)	<0.001	0.711	0.659
	*p-*value		0.194		0.934		0.028			

*P*-values approximated with Wald tests of the fixed effect in a linear mixed-effects model of Box-Cox transformed TIL count.

#### Effect of TLS components on TILs

The unit of analysis are TLS and its proximal tumor nest, defined as tumors within 500 micron × 500 micron field of view (analysis done at the TLS-level). The outcome of interest are TIL counts (Table 4). The primary independent variable is the distinction between *primary stage* TLS and *aggregate stage* TLS. Median, Q1, and Q3 TIL counts are presented. The statistical significance of differences in natural-log-transformed TIL counts between the primary aggregate stage TLS were modeled using Mixed Effect models in the formation described in Laird and Ware (43), with a random intercept defined for each BCC containing TLS (*n* = 19). Furthermore, this analysis was repeated for various subsets of the data, namely at different body sites, gender, age range category, BCC histological subtype and TLS maturation stage, and binned at TLS sizes, T- cell and B-cell compositions. A secondary analysis compared the TIL counts in primary- or aggregate-stage TLS vs. the TIL count in controls which no TLS was identified.

**Table 4:** Effect of TLS properties on TIL count (*n* represents tumor nests either proximal to shared TLS or without TLS in control samples).

		**Primary TLS**		**Aggregate TLS**		**Modeled effect**	**Overall TLS**		**Control**		**Modeled effect**
		***n***	**Median TIL count (IQR)**	***n***	**Median TIL count (IQR)**	***p-*value**	***n***	**Median TIL count (IQR)**	***n***	**Median TIL count (IQR)**	***p-*value**
Total tumor nests		20	46.1 (24,135)	60	33.2 (18,52)	0.110	80	37.5 (20,56)	11	31.8 (28,36)	0.566
**Location**											
	Head and neck	15	42 (24,127)	32	31.3 (15,52)	0.051	47	34.4 (18.9,57)	6	30.8 (20,36)	0.391
	Trunk	3	145 (114,186)	18	28.8 (16,83)	-	21	44 (20,94)	4	33.9 (32,50)	-
	Extremity	2	27.9 (17,39)	10	41.8 (30-49)	-	12	41.8 (25,49)	1	26.8	-
**Gender**											
	Male	17	49.5 (25-132)	50	37.5 (19-52)	0.129	67	41.9 (20-68)	5	35.1 (32,38)	0.727
	Female	2	129 (72,187)	5	26.7 (15,31)	-	7	27 (15,41)	6	30.8 (20,32)	0.921
	Unknown	1	16.2	5	34.4 (30,35)	-	6	32 (20,35)	0	-	-
**Age**											
	43-65	5	83.1 (50,227)	17	27.3 (19,49)	0.559	22	41.8 (19,75)	4	34.7 (26,52)	-
	66-90	14	42.3 (25,129)	38	34.4 (16,52)	0.062	52	39.1 (21,57)	7	31.7 (28,34)	0.63
	Unknown	1	16.2	5	34.4 (30,35)	-	6	32 (20,35)	0	-	-
**Infiltrative component**											
	Absent	13	42.7 (25,132)	51	36.8 (19,52)	0.811	64	40.9 (20,53)	9	31.8 (27,35)	0.454
	Present	7	61.7 (21,154)	9	29.6 (8,35)	0.070	16	32 (16,60)	2	33.8 (32,36)	-
**Nodular component**											
	Absent	2	32.5 (29,36)	1	96.8	-	3	40.1 (33,69)	4	28.4 (22,46)	-
	Present	18	55.6 (23,142)	59	32.1 (17,51)	0.067	77	36.8 (19,55)	7	32.7 (32,36)	0.748
**Superficial component**											
	Absent	14	91.9 (30,176)	36	42.6 (19,60)	0.056	50	47.1 (23,115)	7	31.8 (31,35)	0.378
	Present	6	32.5 (18,47)	24	25 (18,43)	0.829	30	26.1 (17,44)	4	30.9 (22,50)	-
**Ulceration**											
	FALSE	5	145 (83,227)	11	49.4 (25,118)	0.237	16	88.2 (26,147)	1	17.2	-
	TRUE	15	42 (24,92)	49	30.8 (15,50)	0.102	64	33.2 (18,51)	10	32.2 (30,37)	0.857
**TLS Area**											
	0-0.049	3	40.1 (29,41)	19	34.4 (21,52)	–	22	37.2 (20,51)		–	–
	0.05-0.099	3	25.3 (25,44)	22	21.8 (10,41)	–	25	24.8 (11,42)		–	–
	0.10-0.149	4	46.1 (38,68)	11	49.4 (35,74)	–	15	49.4 (35,74)		–	–
	0.15+	10	138 (33,217)	8	36.1 (23,61)	0.128	18	66.4 (22,144)		–	–
**T cell count**											
	0–249	2	29.8 (24,36)	19	29.6 (11,44)	–	21	29.6 (11,42)		–	–
	250–499	4	32.7 (25,43)	13	36.8 (15,47)	–	17	36.8 (20,47)		–	–
	500–999	3	61.7 (52,92)	16	33.3 (18,52)	–	19	38.2 (24,58)		–	–
	1,000+	11	132 (19,206)	12	49.6 (23,123)	0.957	23	83,1 (23,144)		–	–
**B cell count**											
	0–4	0	–	37	32.1 (15,49)	–	37	32.1 (15,49)		–	–
	5–15	2	39.7 (29,51)	16	42.6 (21,65)	–	18	42.6 (19,60)		–	–
	15+	18	46.1 (25,142)	7	27.3 (32,71)	0.484	25	42.7 (23,123)		–	–
**Ratio T:B**											
	0–49	18	42.3 (23,142)	10	31.1 (22,51)	0.247	28	41 (22,93)		–	–
	50–99	2	91.9 (77,107)	17	26.7 (15,52)	–	19	30.8 (17,58)		–	–
	100–149	0	–	13	30.4 (15,42)	–	13	30.4 (15,42)		–	–
	150+	0	–	20	48.1 (22,54)	–	20	48.1 (22,54)		–	–

Significance of fixed effect in Mixed Effect model of the log transformed TIL count, with a random intercept defined for each BCC (*N*= 19).

#### Relationship between tumor killing and TIL counts

The dependent variables are natural-log-transformed TIL counts in tumor nests proximal to TLS while the independent variable is the presence of tumor killing. TLS maturation stage was used as the factor/stratification variable while lesion was the cluster variable, to account for multiple comparisons within a single lesion. One linear mixed-effects model was created for each analysis. The two *p-*values are derived from Wald tests of the fixed effect in the models.

#### Association of fibrillary matrix morphologies with TILs in individual tumors

Correlation of 13 different morphologies of the fibrillary matrix generated by Twombli with TIL counts was performed using Spearman correlation with significance cutoff at *p-*value <0.05. To capture the overall fibrillary matrix architecture in ECM surrounding individual tumors, we performed principal component analysis (PCA) for these 13 measurements of fibrillary morphologies using prcomp function in R. The contribution of individual fibrillary morphologies to principal components (PC1 and PC2) were determined using fviz_contrib function in factoextra package in R to observe which morphologies were responsible for distribution of tumors along PCs. To determine tumors harboring shared fibrillary morphologies, we performed hierarchical clustering on the principal components (HCPC) which generated 3 distinct fibrillary clusters (top 7 PCs encompassing 96.3% of total variance were used to generate fibrillary clusters; HCPC function in FactoMineR package in R). Differences between median TIL counts across fibrillary clusters were determined using two-tailed Mann-Whitney *U* test. To determine fibrillary morphologies significantly associated with distinct fibrillary clusters, we used correlation as distance metric in hierarchical clustering of PCs. Fibrillary clusters were characterized by fibrillary morphologies with significant mean value in a cluster compared to overall mean across all clusters. The *p-*value are derived from F-test in a one-way analysis of variance (assuming the hypothesis of homoscedsticity).

## Results

### Patient characteristics

Thirty lesions from 22 patients (13 males, 8 females) were included in this analysis (Table 1). The age range was 43–90 years. Most lesions were located on the head and neck (*n* = 16) and trunk (*n* = 11). All lesions were previously untreated and unbiopsied. Only primary biopsies were used to investigate TLS. Ulceration was observed in 21 out of 30 lesions. With respect to BCC subtypes, 16 lesions were pure nodular BCCs, 1 was pure infiltrative BCC, 4 were pure superficial BCC while 3 were nodulo-infiltrative BCCs, 5 were superficial early-nodular BCCs and 1 was superficial and nodulo-infiltrative BCC. Given the relatively small dataset and the multiple subtype classes, we analyzed BCC subtype as comprising superficial, nodular or infiltrative components as analysis categories (Table 1).

### Nodular component in BCCs is associated with high prevalence of TLS

**Figure 1:**
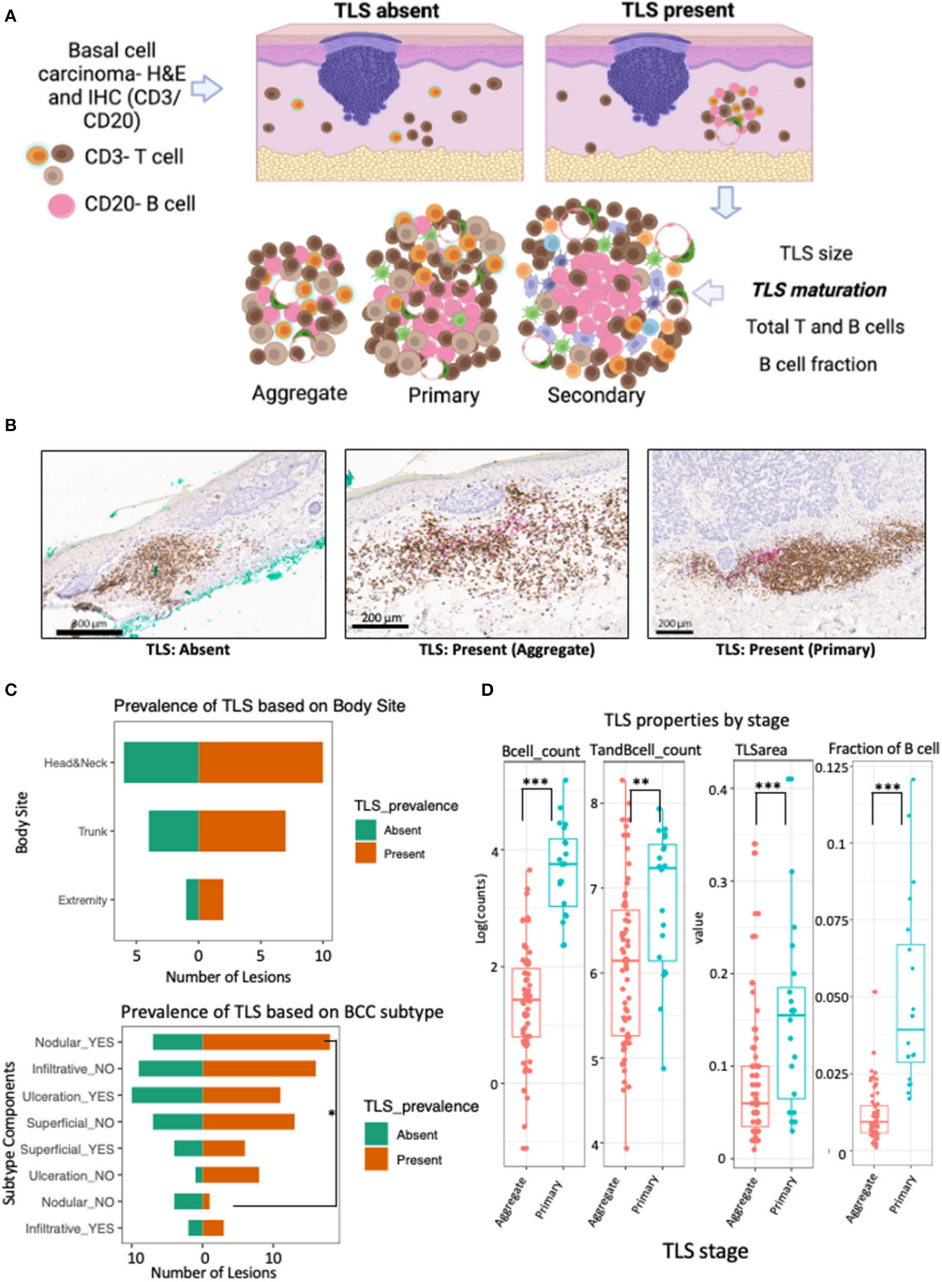
TLS prevalence and maturation depends on BCC subtype and anatomical location. **(A)** Schematic workflow for evaluation of TLS prevalence and maturation. TLS presence was defined as presence and co-localization of both CD3+ T cell and CD20+ B-cell along high endothelial venules in the dermis. TLS maturation was evaluated as dense lymphocytic aggregates composed primarily of T-cells with randomly distributed B-cells (aggregate TLS), CD20+ B-cell clusters with follicular dendritic cells and T-cells (primary TLS) and B-cell clusters showing germinal-center like maturation (secondary TLS). **(B)** Representative images of IHC staining for TLS with CD3+ T cells (brown) and CD20+ B (pink) with patterns indicating absence of TLS when lacking co-localization of T and B cells, presence of aggregate TLS when scattered loose aggregates of T and B-cells are seen in the dermis and presence of primary TLS when CD20+ B-cell initiate cluster formation and are surrounded by CD3+ T cells and dendritic cells. **(C)** Population pyramid displaying prevalence of TLS based on body site (upper panel) and BCC subtype components (bottom panel). A significant over-representation of TLS in lesions with nodular component was observed when compared to lesions without a nodular component (Odds Ratio = 10.29; *p-*value, fisher's exact test = 0.047). No significant difference was detected for TLS prevalence in lesions based on body site, gender, age or ulceration status; although lesions were less likely to have a TLS in the presence of ulceration compared to lesions showing no ulceration (Odds Ratio = 0.14; *p-*value, fisher's exact test = 0.1; see Table 2). **(D)** Box and whiskers plot showing the effect of TLS properties in different maturation stages (aggregate vs primary). Each data point represents individual TLS. More mature primary TLS show a greater amount of B cells (p-value for B cells <0.001), greater number of combined T and B cells (*p-*value = 0.006), larger TLS area (*p-*value = 0.001), and higher fraction of B cells compared to aggregate stages (*p-*value <0.001). *P-*value is the significance of the model coefficient using mixed effect model with random intercept for BCC and fixed effect for TLS maturation stage. Created with BioRender.com.

In this lesion-level analysis, TLS were annotated and evaluated for quantitative features and maturation stage (Figures 1A,B). TLS were found in 19 of the 30 lesions from 16 patients. Importantly, no secondary TLS maturation stages were identified in this dataset. No significant differences in TLS prevalence based on sex, age, or body site was discovered (Table 2; Figure 1C).

A significant presence of TLS was noted in BCC lesions with a nodular component compared to lesions without nodular component (Odds Ratio = 10.29, *p-*value = 0.047; Figure 1C; Table 2). While not significant, lesions with ulceration notably showed a lower probability of TLS presence compared to lesions without ulceration (Odds Ratio = 0.14, *p-*value = 0.1; Table 2; Figure 1C).

To demonstrate cellular composition of TLS were indicative of qualitative maturation staging, we quantified TLS properties in aggregate and primary TLS. Indeed, higher B-cell counts (aggregate median log count = 1.4, primary median log count = 3.8, *p-*value <0.001), higher combined T and B cell counts (aggregate median log count = 6.1, primary median log count = 7.2, *p-*value = 0.006), greater TLS area (aggregate mm^2^ = 0.06, primary mm^2^ = 0.155, *p-*value = 0.001), and higher fraction of B cells (aggregate median = 0.009, primary median = 0.039, *p-*value <0.001) were all significantly strong predictors of a more mature (primary) TLS stage (Figure 1D).

### Primary maturation stage of TLS corresponds with higher TIL counts as compared to aggregate TLS

**Figure 2:**
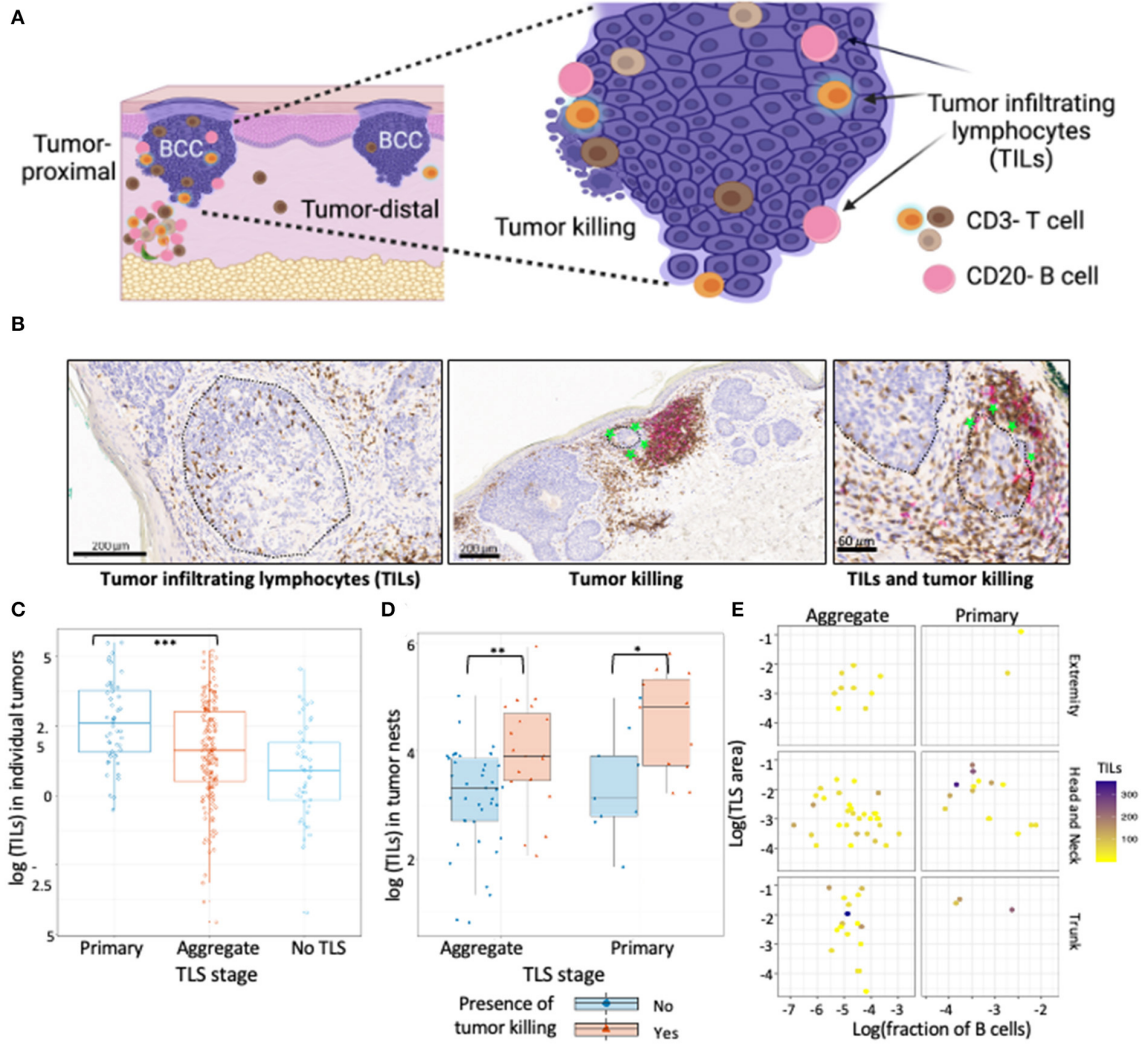
TILs in BCC show association with TLS presence and maturation. **(A)** Schematic representation of tumor location with respect to TLS, TILs and tumor killing in BCC tumors. Proximal tumors were defined as adjacent to TLS, distal tumors were located farthest from the TLS. Infiltrating T and B cells inside annotated tumor boundaries were quantified and referred to as TILs. Tumor killing was defined as active tumor regression involving a combination of infiltrating immune cells along tumor periphery leading to disruption of the palisaded architecture and was graded as a binary variable for presence or absence. **(B)** Representative CD3/CD20 IHC images of TILs and tumor killing demonstrating CD3+ T cells (brown) and CD20+ B cells (pink) cells inside tumors (black encircled structures). Infiltrating CD3+ and CD20+ lymphocytes inside BCC tumors were referred to as TILs while infiltrating of immune cells along tumor edge that accompany a disrupted tumor border was referred to as tumor killing (green stars). **(C)** TILs distribution in tumors proximal to primary TLS, aggregate TLS and control cases (no TLS); data points represent individual tumors. Tumors proximal to primary TLS show significantly higher mean of TIL counts compared to tumors proximal to aggregate TLS (*p-*value < 0.001, approximated with Wald tests of the fixed effect in a linear mixed-effects model of Box-Cox transformed count of TILs, see Table 3). **(D)** Abundance of TILs in tumor nests proximal to TLS denoted for presence of tumor killing. Presence of tumor killing is associated with higher TIL counts for tumor nests proximal to primary TLS (*p-*value = 0.029) and similarly for tumor nests proximal to aggregate TLS (*p-*value = 0.01). *P-*values derived from Wald test for fixed effects in linear mixed-effect models. **(E)** Observation of TILs distribution with combined effect of TLS properties, TLS stage and body site; data point represents tumor nests. No significant difference in median TILs across TLS stage (primary vs. aggregate) was observed for individual TLS properties, body site determined by mixed effect model of log transformed TILs counts with random intercept defined for each BCC lesion (see Table 4). Created with BioRender.com.

To determine the effect of TLS maturation stage on anti-tumor immunity, we documented the level of TILs along with extent of tumor killing with respect to proximity of tumor to TLS (Figures 2A,B).

Tumors proximal to primary TLS displayed significantly higher TIL counts compared to tumors proximal to aggregate TLS (Figure 2C; Table 3). Higher TIL counts were associated with tumor killing irrespective of TLS stage (Figure 2D). Multiple TLS in each lesion may be suggestive of a locally effective range for each TLS, thus we investigated differences in TIL counts between lesion-matched proximal and distal tumors in a subset of BCCs (Supplementary Figure S1A). In these 5 BCCs, 3 showed the anticipated relationship of higher TIL counts in tumors proximal to TLS compared to distal tumors. Conversely, 2 BCCs showed higher or comparable TIL counts in distal tumors compared to proximal tumors. Although no significant difference in TIL counts was observed across proximal vs. distal tumors in primary or aggregate TLS maturation stages, heterogeneity in TIL counts was apparent on this small subset, which may be due to factors beyond TLS. In fact, presence of mucin emerged as a potential contributor to heterogeneity when assessing peri-tumor stroma in patients which displayed higher TIL counts in distal tumors (Supplementary Figure S1B). Large quantities of secreted mucin in the peritumoral region seem to have served as physical barriers to immune infiltration in proximal tumors, indicating additional stromal factors likely modulate lymphocytic infiltration in tumors.

We next investigated the influence of individual TLS components on TIL counts in tumor nests proximal to TLS and discovered no significant differences in median TIL counts between primary and aggregate maturation stages (Table 4). While individual bivariate comparisons of TLS components showed no significant relationship with the outcome of TIL counts, we appreciated a combined effect of these factors likely impacted TIL counts. Despite lacking the statistical power to perform a multivariate analysis, we explored TIL count distribution in proximal tumor nests by plotting a combination of TLS features (maturation stage, body site, TLS area, and fraction of B-cells). Overall, tumor nests proximal to TLS with greater area and/or higher fraction of B-cells displayed higher TIL counts. Given the small sample sizes for lesions on extremity and trunk, we only focused on lesions located on the head and neck and observed greater TIL counts in tumor nests proximal to primary TLS as compared to aggregate TLS. However, a great amount of variability in TIL counts persisted within TLS maturation stages (Figure 2E). This suggested additional features within the tumor microenvironment, such as ECM components, contributed to modulating lymphocytic infiltration in tumors, which was subsequently studied.

### Distinct tumor-associated fibrillary matrix morphologies explain heterogenous distribution of TIL counts

**Figure 3:**
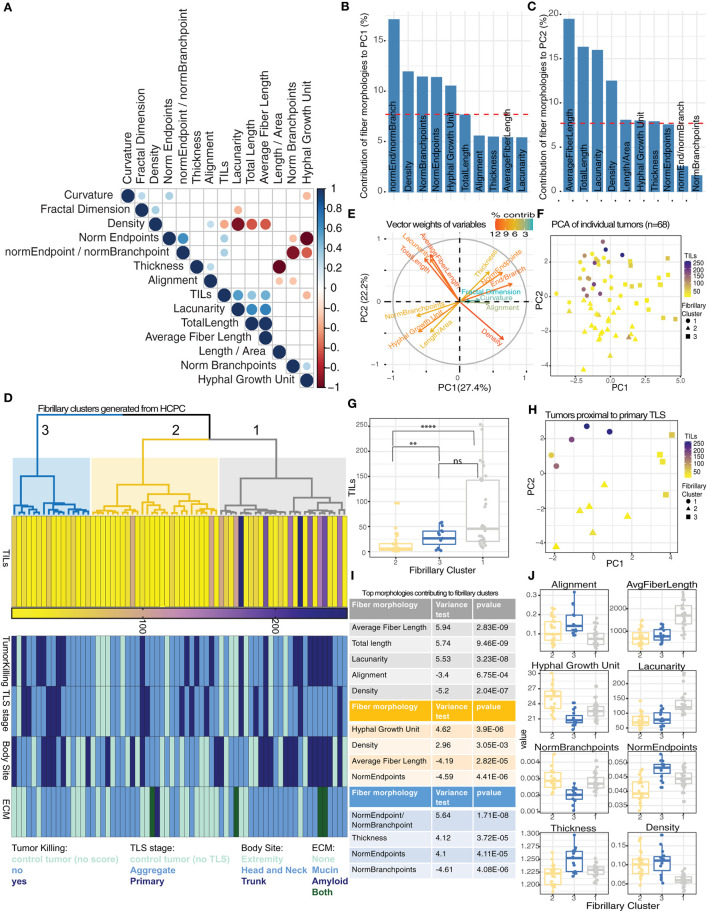
Discrete morphologies in tumor-associated fibrillary matrix impact variation in TIL counts. **(A)** Correlation matrix reporting significant relationships (Spearman correlation, *p-*value < 0.05) among 13 fibrillary matrix morphologies and TIL counts for 68 tumors. **(B,C)** Barplot showing contribution of top 10 fiber morphologies to PC1 **(B)** and PC2 **(C)** from PCA. Dashed red line corresponds to expected value if contribution were uniform indicating morphologies exceeding this value are important contributors to PCs. **(D)** Dendrogram from HCPC shows 3 clusters of tumors based on similarity in fibrillary morphologies (gray = cluster 1, yellow = cluster 2, blue = cluster 3). Heatmaps reporting TIL counts, Tumor killing, TLS maturation stage, body site, and ECM components for each tumor distributed across fibrillary clusters. **(E)** Vector weights for individual morphologies contributing to separation of samples along PC1 and PC2 (color indicates percent contribution). **(F)** Scatterplot using dimensions from PC1 and PC2 generated from PCA summarizing fibrillary matrix morphologies for individual tumors. TIL counts are indicated by color gradient (purple = high, yellow = low) and shape indicates fibrillary cluster assigned based on hierarchical clustering of PCs. **(G)** Boxplot displaying median TIL counts for individual tumors based on fibrillary cluster. Distribution of TIL counts were significantly higher in fibrillary cluster 1 compared to cluster 2 (*p-*value < 0.0001) and higher in cluster 3 compared to cluster 2 (*p-*value = 0.009), while no significant difference was reported between cluster 1 and 3; Two-tailed Mann-Whitney U. **(H)** Scatterplot using dimensions from PC1 and PC2 generated from PCA summarizing fibrillary matrix morphologies for tumors proximal to primary TLS. TIL counts are indicated by color and shape indicates fibrillary cluster. **(I,J)** Table (i) and boxplots (j) reporting fiber morphologies significantly associated with each fibrillary cluster resulting from HCPC. Square correlation coefficient and *p-*value of the *F*-test in a one-way analysis of variance is reported in the table (i).

To address the variability in TIL counts for individual tumors proximal to distinct TLS stages, we investigated the diversity of fibrillary morphologies in the ECM immediately surrounding tumors (Supplementary Figures S2A–D). We observed significant correlations between TIL counts and morphologies that described individual fibers (such as number of endpoints and fiber length) in addition to global patterns of the matrix, such as density and lacunarity (Figure 3A). Higher TIL counts were associated with increased lacunarity and fiber length whereas reduced TIL counts were associated with increased hyphal growth unit and increased high density matrix values (Supplementary Figures S3A–D). To summarize all 13 morphologies for individual tumor-associated fibrillary matrices we performed principal component analysis (PCA). The first two principal components (PCs) captured 49.3% of the total variability in fibrillary morphologies across all tumors (Supplementary Figure S3E). Tumors distributed along PC1 differed largely in measurements for endpoints/branchpoints, density, number of branchpoints, number of endpoints and hyphal growth unit (Figure 3B), whereas tumors distributed along PC2 displayed variation in average fiber length, total length, lacunarity and density (Figure 3C). To identify groups of tumors exhibiting similar measurements of fibrillary morphologies, we performed hierarchical clustering on principal components (HCPC), which generated 3 distinct clusters (Figure 3D). Critically, tumors within fibrillary cluster 1 showed significantly higher TIL counts compared to cluster 2 (Figures 3D,F,G) and were characterized by morphologies positively contributing to PC2, such as average fiber length and lacunarity, along with morphologies negatively contributing to PC1, such as density (Figures 3E,F). While variation in fibrillary morphologies explained variation in TIL counts across individual tumors, these morphologies did not distinguish tumors exhibiting variation in tumor killing nor did they align with presence of other important features of the ECM such as amyloid or mucin. Additionally, fibrillary clusters showed no pattern in body site or TLS maturation stage (Figure 3D; Supplementary Figures S3F,G).

Since TLS maturation stages were randomly distributed across all three fibrillary clusters, we investigated whether the variability in TIL counts within a particular TLS maturation stage (or control group) could be explained by variability in fibrillary morphologies. For tumors proximal to primary TLS, only those within fibrillary cluster 1 displayed higher TIL counts (Figure 3H). This same pattern of TIL counts was observed for tumors proximal to aggregate TLS as well as control tumors with no TLS (Supplementary Figures S3H,I). These results highlight the importance of fibrillary matrix architectures in facilitating lymphocytic infiltration and attributes morphologies specifically from cluster 1 with supporting a conducive tumor microenvironment for TILs.

To interrogate defining characteristics of each distinct fibrillary cluster, we compared the differences in means for each morphology in a particular cluster compared to the overall mean across all clusters. We found an increase in average fiber length, total length and lacunarity were all significantly associated with fibrillary cluster 1. Additionally, decreased alignment and reduced density were significantly associated with fibrillary cluster 1 (Figures 3I,J). Meanwhile, fibrillary cluster 2 was characterized by a significant increase in hyphal growth unit and density along with reduced average fiber length and numbers of endpoints (Figures 3I,J). Comparing morphologies across fibrillary clusters suggested environments which are either conducive for or hindering anti-tumor immunity. Overall, neither TLS nor fibrillary matrix morphologies alone adequately determined effective behavior of TILs. Here we demonstrated preliminary effects of altered and diverse fibrillary matrix morphologies in modulating TLS function by creating permissive or restrictive conditions for tumor infiltration.

## Discussion

In this study, we investigated the prevalence of TLS, their association with TILs and tumor killing and the influence exerted by fibrillary morphology in the ECM on the prognostic value of TLS in BCCs. BCCs are the most common cancer globally (44). BCCs are locally invasive, relatively slow-growing tumors that are associated with high survival rates (45). Depending on BCC subtype, treatment can include Mohs surgery, electrodesiccation and curettage, photodynamic therapy and topical immunotherapy using toll-like receptor agonist, Imiquimod (46). BCCs demonstrate both immunosuppressive and anti-tumor states in a dynamic equilibrium. When this equilibrium is disrupted, tumor progression or regression result (47, 48). In fact, BCCs are known to undergo immune-mediated spontaneous regression (39). Thus, BCCs are an interesting model to study the prevalence of TLS and the impact of ECM features on anti-tumor immunity. Further, by acquiring an understanding of additional predictors of prognosis, better stratification for treatments can help streamline management of BCCs. In this study, we used dual IHC for CD3^+^ and CD20^+^ to analyze and quantify TLS quantify TILs and corresponding H&E tissue section to quantitate ECM morphology in BCC tumors.

In BCCs, TLS prevalence was similar across all anatomical locations, although sun-exposed locations such as the head and neck were found to have more mature TLS stages (primary TLS). Further, more TLS were found in older individuals (>65 years), and in males. Evidently, when controlling within a patient with multiple BCCs, higher TLS prevalence and maturation was found in the sun-exposed anatomical locations, i.e., head and neck as compared to trunk. Since sun-exposure and UV-irradiation can lead to immunosuppression, fewer or immature TLS would have been expected on sun-exposed sites (49). However, cumulative sun-exposure also leads to higher number of UV-induced mutations and consequently a higher tumor mutational burden (TMB) and neoantigen production (50). TMB and neoantigens positively correlate with TLS density in multiple cancer types (51) and associate with robust immune responses, thus potentially explaining the increased TLS maturation. As seen in other solid tumors, impact of UV-induced mutational burden and effect on TLS maturation will need to be correlated with TLS by analyzing TMB, UV-specific mutational signatures and expression of DNA repair enzymes such as PARP1 in BCC tumors (52). Increased TLS prevalence in males will be analyzed in more balanced cohorts in future since a single male patient contributed multiple lesions (*n* = 5) in this analysis. Additionally, we only found aggregate and primary maturation stages of TLS in BCC, no secondary maturation stage of TLS was found. Our results on primary BCCs align with the studies in melanoma where very few secondary TLS were found in primary melanoma biopsies as opposed to metastatic tissues (11). In another study on cSCC, majority of cases had mature TLS while only few showed immature TLS when evaluated using a 2-stage maturation class (12).

Primary TLS was associated with comparatively higher TIL counts and tumor killing as compared to aggregate TLS and no TLS. More mature TLS implies increased generation of effector T cells and plasma cells with the potential to control cancer growth and dissemination. These findings have been mirrored in other cancers (36). Some distal and control tumors were also found to have moderately high TIL counts. This could be attributed to several factors. In few pigmented BCCs, melanin (brown pigmentation on H&E and IHC) caused significant contamination of positive signals (similar to T-cells) on IHC leading to inflated TIL counts in control tumors. Further, in some tumors, presence of peritumoral mucin seems to have precluded infiltrating cells from entering tumors (Supplementary Figure S1) leading to comparatively higher TIL counts in distal tumors devoid of peritumoral mucin. Secreted mucins can exert both immunomodulatory roles, through physical barriers and modulating immune activation and function (53). To truly understand presence of higher TIL counts in distal tumors, spatial significance and local effects of TLS need to be investigated in the presence of specific stains for mucin and amyloid on entire tissue sections.

Individual relationships between fiber morphologies and TILs provide important explanations for how alterations in the ECM may impact immune cell behavior in the tumor microenvironment. For example, an increase in lacunarity was associated with an increase in TIL counts whereas an increase in matrix density was negatively associated with TILs. Lacunarity describes the amount of space between fibers thereby indicating a fibrillary matrix with more ‘gaps' between fibers allows for a more permissible environment for tumor infiltrating lymphocytes to access tumor. In contrast, a matrix that is denser in fiber composition restricts access of immune cells to tumor. While individual fiber morphologies were significantly correlated with TILs, no single fiber morphology fully explains variability in TILs across tumors within a particular TLS stage group. Therefore, we performed hierarchical clustering on principal components from measurements across 13 different fiber morphologies. This approach allowed us to identify the combined effect of fibrillary matrix morphologies contributing to variation in TIL counts, where tumors confined to fibrillary cluster 1 displayed the greatest level of TILs regardless of TLS stage. Fiber morphologies associated with tumors displaying significant differences in TIL counts indicate an immune-modulating behavior which enhances (fibrillary cluster 1) or impedes (fibrillary cluster 2) immune cell access to tumor, which agrees with other studies showing these same features are prognostic markers in breast cancer, oral squamous cell carcinoma, gastric carcinoma and pancreatic cancer to name a few (22, 54–57). For example, in breast cancer, more linear and aligned fibers have been associated with a more aggressive cancer shown through invasiveness of cancer cells (22, 55, 58). Interestingly, the fibrillary clusters largely explaining variation in TILs across tumors did not resolve tumor killing phenotype, suggesting that while fibrillary morphology is one of the important contributors, the process of immune infiltration leading to tumor killing is multifactorial and regulated by additional cues within the tumor microenvironment. Overall, our study emphasizes the importance of considering fibrillary matrix morphologies as a critical feature when assessing TLS impact on TILs and tumor killing.

There is growing evidence that role of ECM components extend beyond physical support and/or barrier to cell types within the tissue and also serve immunomodulatory role (21). Several studies have shown that various collagen types and other ECM proteins are capable of directly modulating immune activity (13). Additionally, stromal cells, largely fibroblasts, known to deposit ECM components and dynamically respond to changing environments, have recently been appreciated as immune modulators capable of transitioning into altered cellular states which either repress or promote tumor cell survival; referred to as cancer associated fibroblasts (CAFs) (59). Linking the spectrum of cellular states within the fibroblast population to accompanying changes in ECM extending to its effect on immune cell behavior and outcomes on cancer cell survival will be critical to improve our ability to predict patient response to therapy, expanding our current understanding of TLS prognostic value.

There were several limitations associated with the study. This study relied only on two immune cell markers (CD3^+^ T-cell and CD20^+^ B-cell) to investigate TLS prevalence, maturation, and function. To understand this phenomenon further would require staining for additional immune cell subsets that mediate immune-activation and immune-suppression in skin such as Langerhans cells, macrophages and mast cells. Future work could examine intratumoral B cells, plasma cells and TLS as potential biomarkers of patient prognosis and response to immunotherapies. Spatially resolved global approaches, such as spatial transcriptomics, can potentially uncover novel spatial ECM features within TME that can interact with known players such as collagen, elastin, mucin and amyloid to modulate anti-tumor immunity. Further, the analysis was performed using paired sections for IHC and H&E (1 section for each). Since the analysis was done on single 2D sections, we defined tumor and stroma boundaries based on 2D space. We recognize the loss of 3D organization and evolution of these dynamic structures and consequently our constrained ability to detect and score TLS maturation. Additionally, since the analysis was performed on distinct IHC and H&E sections, correlation of TILs in IHC with ECM morphologies on H&E should be interpreted with caution, since tumor and stroma margins change between sections.

We confirm the prevalence of aggregate and primary TLS in BCCs, TLS association with TILs and demonstrate the importance of fibrillary matrix architectures in facilitating lymphocytic infiltration into tumors. While our results suggest that fibrillary morphology is one of the important contributors, the process of immune infiltration leading to tumor killing is multifactorial and regulated by additional cues within the tumor microenvironment that should be accounted to improve prognostic value of TLS.

## Data availability statement

The raw data supporting the conclusions of this article will be made available by the authors, without undue reservation.

## Ethics statement

The studies involving human participants were reviewed and approved by MSKCC-IRB. The patients/participants provided their written informed consent to participate in this study.

## Author contributions

Study conception and design: CB, MG, NK, SG, CA-F, MC, VR, C-SC, AM, PG, JD, KK, MR, and AS. Data collection: CB, MG, KK, MH, and AS. Analysis and interpretation of results: CB, MG, NK, MH, SG, CA-F, MC, VR, JD, PG, AM, C-SC, KK, MR, and AS. Draft manuscript preparation: CB, MG, MR, and AS. All authors contributed to the article and approved the submitted version.

## Funding

This work was funded by NIH/NCI Cancer Center Support Grant P30 CA008748 (MSKCC), NIH/NIBIB R01EB028752 and NIH/NCI 240771 (MR), Melanoma Research Alliance (AS), Roux Institute (CB), Harold Alfond Foundation (CB), The Institute for Experiential AI (CB), and National Center for Advancing Translational Sciences (NCATS) grant 2 KL2 TR0002385-06 of the Clinical and Translational Science Center at Weill Cornell Medical College (VR).

## Conflict of interest

Author MG is a consulting investigator for DBV technologies; research consultant: Dermatology Service, MSKCC. Author CA-F consultant for and owns equity in Caliber I.D., manufacturer of the VivaScope RCM. Author AM is an honorarium for dermoscopy lectures (3GEN), royalties for books/book chapters, dermoscopy equipment for testing, payment for organizing and lecturing (American Dermoscopy Meeting). Author C-SC had research funding from Apollo Medical Optics, Inc. Author MR was employee of and owns equity in Caliber I.D. VivaScope is the commercial version of a laboratory prototype he developed at Massachusetts General Hospital, Harvard Medical School. Author PG was employed by Melanoma Institute Australia. Author VR is an expert consultant for Inhabit Brands, Inc. The remaining authors declare that the research was conducted in the absence of any commercial or financial relationships that could be construed as a potential conflict of interest.

## Publisher's note

All claims expressed in this article are solely those of the authors and do not necessarily represent those of their affiliated organizations, or those of the publisher, the editors and the reviewers. Any product that may be evaluated in this article, or claim that may be made by its manufacturer, is not guaranteed or endorsed by the publisher.
