# Supplementary material for: Tertiary lymphoid structures accompanied by fibrillary matrix morphology impact anti-tumor immunity in basal cell carcinomas
Source: Front Med (Lausanne). 2022 Oct 27;9:981074. doi: 10.3389/fmed.2022.981074 (PMC9647637; doi:10.3389/fmed.2022.981074)
Supplement: Supplementary file 1 [file Data_Sheet_1.pdf]

Supplementary Figures:

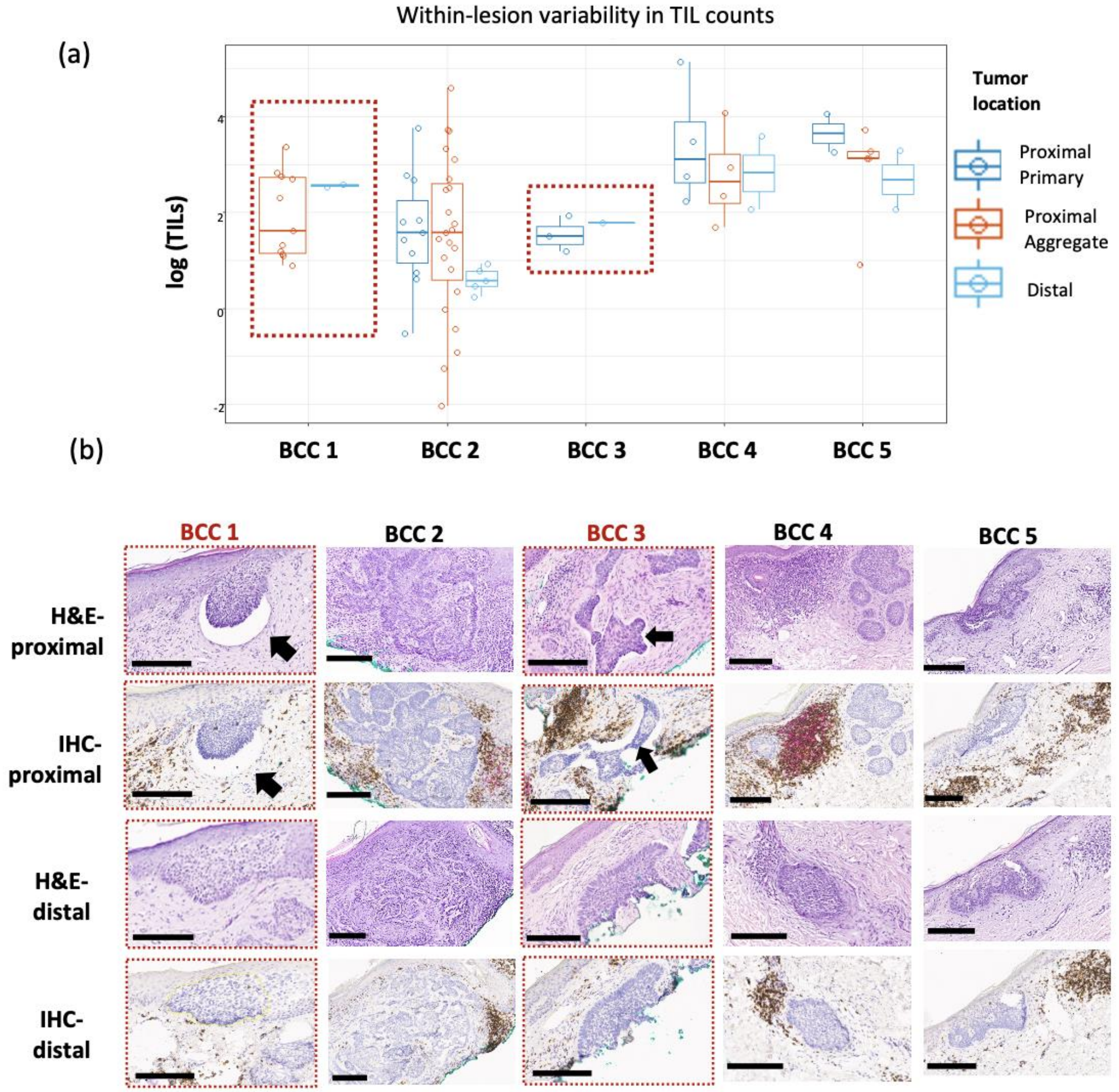

Figure S1. Spatial relationship and heterogeneity between TILs associated with primary TLS, aggregate TLS and distal tumors in same patients.

1a) Box plots displaying TIL counts for individual tumors grouped by proximity to primary or aggregate TLS. Only 5 BCCs harbored both proximal and distal tumors in both H&E and corresponding IHC sections allowing for interrogation into spatial significance of TLS. Using a general linear model with BCC lesion as covariates, no significant differences in log-transformed TIL counts was observed for tumors proximal to primary TLS compared to tumors proximal to aggregate TLS (p-value = 0.413) or tumors proximal to primary TLS compared to tumors distal to TLS (p-value = 0.205). Among these 5

BCCs, 3 (lesions 2, 4 and 5) show proximal tumors with higher median TIL counts compared to lesion matched distal tumors. Whereas 2 BCCs (lesions 1 and 3 outlined in red) show higher TIL counts in distal tumors compared to proximal tumors. This highlights, in a small subset of samples, location of proximal tumors alone did not influence TIL count.

1b) Representative images from H&E and corresponding IHC section (brown = CD3+ T cells, pink = CD20+ B cells) BCC 1-5. BCC 2, 4 and 5 show expected relationship of higher TIL counts for proximal tumors compared to distal tumors. However, BCC 1 and 3 show higher TIL counts for distal tumors compared to proximal tumors within the same lesion. Binary assessment of presence or absence of mucin was quantified using paired H&E sections to relate to TIL counts in IHC sections. BCC 1 and 3 show presence of mucin surrounding tumors proximal to TLS (arrows) with observed reduction in TIL counts compared to lesion matched distal tumors. BCC 2, 4 and 5 show no evidence of mucin at periphery of tumor boundary for tumors proximal to TLS coinciding with higher TIL counts compared to lesion matched distal tumors.

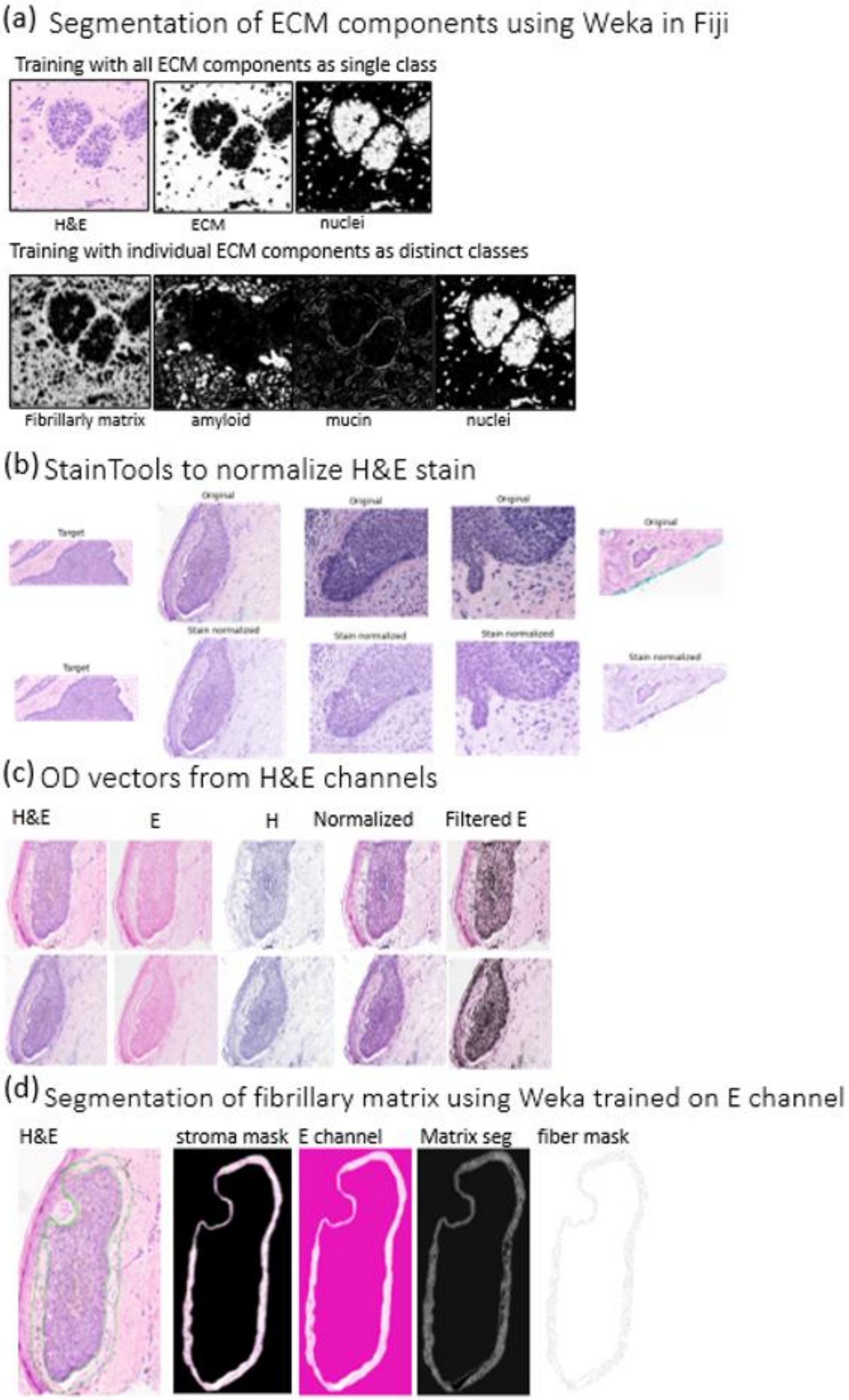
(a) Segmentation of ECM components using Weka in Fiji
Training with all ECM components as single class
H&E
ECM
nuclei
Training with individual ECM components as distinct classes
Fibrillarly matrix
amyloid
mucin
nuclei
(b) StainTools to normalize H&E stain
Target
Original
Stain normalized
(c) OD vectors from H&E channels
H&E
E
H
Normalized
Filtered E
(d) Segmentation of fibrillary matrix using Weka trained on E channel
H&E
stroma mask
E channel
Matrix seg
fiber mask

Figure S2. Development of image processing pipeline to measure tumor-associated fibrillary matrix morphologies

A. Examples for two different approaches to train Weka segmentation model in Fiji to segment components of the ECM and nuclei from H&E histology sections. Top panel describes segmentation when using a single class for ECM encompassing fibrillary matrix, amyloid and mucin and another class for nuclei. Bottom panel describes improved segmentation when using discrete classes for ECM components.
B. Normalization approach for image processing input for Weka segmentation. H&E images normalized using StainTools to color correct images with sub-optimal contrast between ECM and nuclei. Top panel shows original images with target sample representing good contrast in H&E serving as reference in color correction for poorer quality images. Bottom panel shows result of color normalization showing minimal improvement in recovering contrast comparable to target image.
C. Alternative approach for processing samples for input in Weka segmentation model using OD vectors from H&E channels. OD vectors from ideal H&E images (top panel) were used to deconvolute eosin from hematoxylin channel and identify OD space to convert sub-optimal values (bottom panel) to OD space from ideal images. Resulting normalized images were further filtered to remove hematoxylin signal (black denotes hematoxylin signal removed in resulting filtered eosin image). Improved quality in ECM contrast with nuclei was shown in normalized images using OD vectors compared to StainTools approach above.
D. Final approach for generating fiber masks used in measuring fibrillary matrix morphologies. H&E images were annotated for tumor region and surrounding stromal region (green annotation in H&E image in far-left panel; stroma annotation was marked 30 µm from tumor boundary). Using manual annotations on H&E images, a stroma mask was generated using Seg3D to remove tumor region. Next, H&E deconvolution was performed in Fiji and the resulting eosin channel image was used as input in Weka segmentation plugin in Fiji (trained on eosin images for segmentation of distinct classes for ECM and nuclei). After segmentation, probability maps for fibrillary matrix class were then used as input for generating fiber masks to measure fiber morphologies using Twombli macro in Fiji.

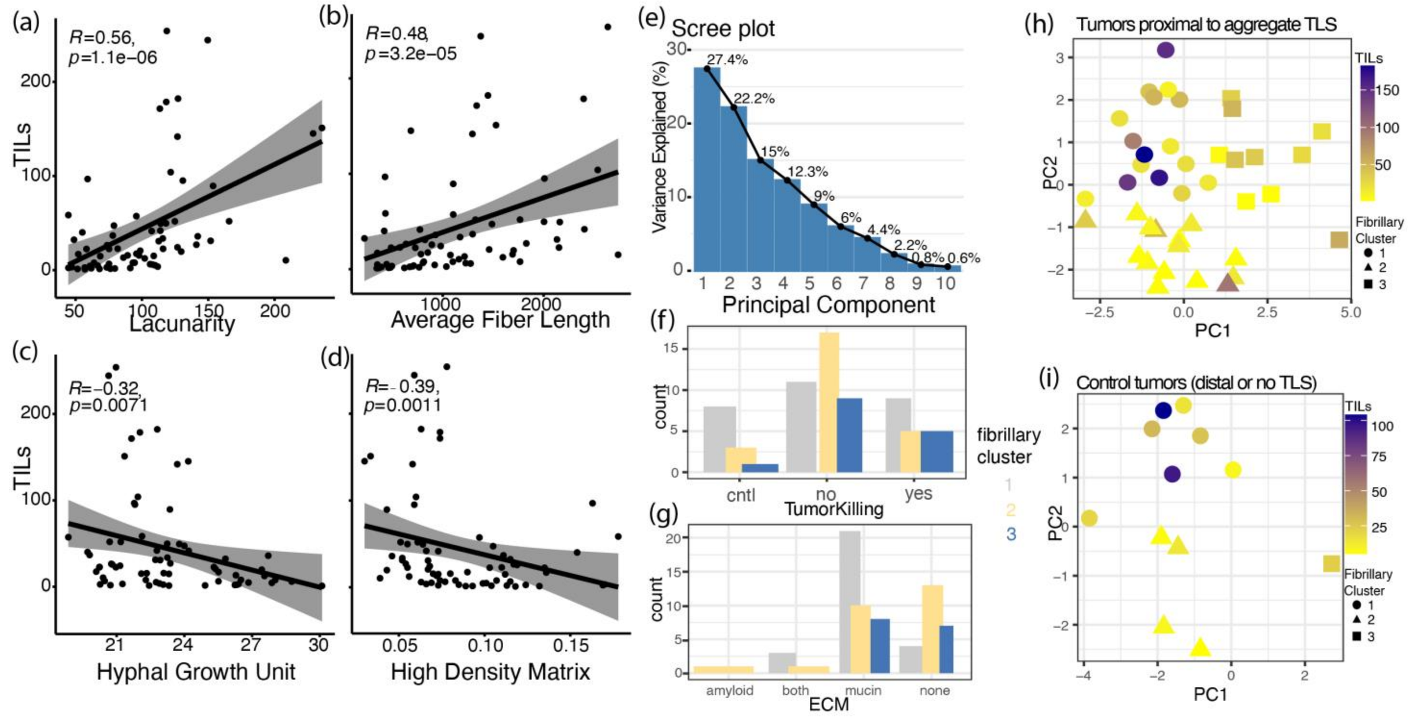

Figure S3. **Multifactorial contribution of ECM components underly heterogeneity in TIL counts**

A – D. Scatterplots for significant correlations (Spearman, p-value < 0.05) between TIL counts and fibrillary matrix morphologies in 68 individual tumors. Lacunarity (a) and average fiber length (b) were positively correlated with TIL counts whereas hyphal growth unit (c) and density (d) were negatively correlated with TIL counts.

E. Scree plot showing percentage contribution to variance for each first 10 principal components (PC).

F&G. Barplot for distribution of tumors within fibrillary clusters (1 = gray, 2= yellow, 3 = blue) based on tumor killing status (f; cntl = no score assigned, no = absence of killing, yes = presence of killing) and presence of ECM components (g).

H&I. Scatterplot using dimensions from PC1 and PC2 generated from PCA summarizing fibrillary matrix morphologies for individual tumors proximal to aggregate TLS (h) or control tumors (i). TIL counts indicated by color gradient (purple = high, yellow = low) and shape indicates fibrillary clusters.
